# Nonsurgical Treatment for Vocal Fold Leukoplakia: An Analysis of 178 Cases

**DOI:** 10.1155/2017/6958250

**Published:** 2017-06-14

**Authors:** Min Chen, Lei Cheng, Chang-jiang Li, Jian Chen, Yi-lai Shu, Hai-tao Wu

**Affiliations:** ^1^Department of Otolaryngology-Head and Neck Surgery, Eye and Ear, Nose, Throat Hospital of Fudan University, Shanghai, China; ^2^Shanghai Key Clinical Disciplines of Otorhinolaryngology, Shanghai, China

## Abstract

**Objective:**

To assess the effectiveness and identify vocal fold leukoplakia types appropriate for nonsurgical treatment.

**Methods:**

The vocal fold leukoplakia in 178 patients was divided by gross appearance into three subtypes: flat and smooth, elevated and smooth, and rough. All patients received nonsurgical treatment including smoking and drinking cessation, voice rest, omeprazole, and Chinese medication therapy. The clinical response of three subtypes was assessed after a 6-month follow-up.

**Results:**

Vocal fold leukoplakia subtypes included flat and smooth (*n* = 66; 37.1%), elevated and smooth (*n* = 103; 57.9%), and rough (*n* = 9; 5.0%). The rate of complete response was 80.3%, 66.0%, and 0.0% for the 3 lesion types, respectively (rough versus flat and smooth, *P* < 0.001; rough versus elevated and smooth, *P* < 0.001, Fisher's exact test). The incidence of carcinoma in rough leukoplakia was significantly higher than that in smooth leukoplakia (44.4% versus 2.4%, *P* = 0.002, Fisher's exact test). Clinical type was the only significant factor for clinical response of nonsurgical treatment (*P* = 0.005, ordinal logistic regression).

**Conclusions:**

The effectiveness of nonsurgical treatment for smooth vocal fold leukoplakia is better in comparison to rough vocal fold leukoplakia. Smooth leukoplakia could be managed with nonsurgical treatment; more aggressive treatments should be considered for rough leukoplakia.

## 1. Introduction

Vocal fold leukoplakia is clinically defined as white mucosal lesions that cannot be characterized as any other condition and is pathologically divided into two subtypes as follows: keratosis with nondysplasia and keratosis with dysplasia [1, 2]. Leukoplakia without dysplasia does not convey premalignant potential and leukoplakia with dysplasia demonstrates premalignant potential [3]. However, benign and malignant lesions of vocal leukoplakia could not be discriminated clinically without a pathological biopsy; therefore, a consensus treatment strategy ranging from observation to complete resection for vocal fold leukoplakia has not been reached [4].

Vocal fold leukoplakia should be treated individually according to its benign or malignant possibility. A conservative therapy or observation strategy benefits those with a low risk of malignancy [1, 5]. To data, surgical therapy remains the most widely studied modality of treatment. Ricci and Isenberg reported that approximately 50% of patients with clinical diagnosis of vocal fold leukoplakia do not have dysplasia [6, 7], indicating that these patients received unnecessary surgical treatment.

Considering the macroscopic appearance, a classification and staging system of oral leukoplakia has been proposed [8]. However, besides a laryngoscopic imaging scoring system established by Fang et al. [9], there have been few reports about the clinical classification method of vocal fold leukoplakia to distinguish benign from malignant lesions. Thus, a method to classify the vocal fold leukoplakia can reflect the degree of lesions simply and comprehensively might be indispensable.

Some studies evaluated the effectiveness of nonsurgical intervention for oral leukoplakia [10, 11], showing a significant effect of nonsurgical therapy. However, it is still unclear whether patients with vocal fold leukoplakia can benefit from nonsurgical treatment. Most studies have focused on the surgical treatment but ignored the nonsurgical treatment for vocal fold leukoplakia.

The purpose of this study was to propose a new classification method to vocal fold leukoplakia and to assess the clinical response of nonsurgical treatment in order to optimize treatment strategies.

## 2. Material and Methods

The protocol of this study was approved by the Institution Review Board of the Eye and Ear, Nose, Throat Hospital of Fudan University, Shanghai, China.

### 2.1. Patients

Clinical data of 604 outpatients with a primary diagnosis of vocal fold leukoplakia from January 2010 and December 2014 were reviewed. Patients scheduled for nonsurgical treatment were included in this study. The clinical diagnosis of the leukoplakia was confirmed by three experienced laryngologists according to medical history and laryngoscope examination. Any other specific disorders that could appear as a white lesion of vocal cord, such as upper respiratory tract infections, laryngeal tuberculosis, and laryngeal fungus infection, were excluded. Patients who had a respiratory infection history during last two weeks, previous or current tuberculosis infection, or long-term steroids use were excluded. Patients pathologically diagnosed with laryngeal squamous cell carcinoma or who had underwent surgery or radiotherapy of the larynx were also excluded.

### 2.2. Clinical Data

Clinical data including gender, age, smoking history, alcohol consumption, laryngopharyngeal reflux, voice abuse, medication history, laryngoscopic images of pretreatment and posttreatment, and postoperative pathologic records were collected. Smoking was defined as smoking of more than 10 cigarettes each day for at least 1 year. Drinking was defined as consumption of more than 80 mL of pure alcohol per day. Cases regarded as voice abusers met at least one of the criteria below: (1) phonation time that was at least 4 hours per day and (2) professional voice users (such as teachers, anchors, telemarketers, salespeople, instructors, singers, and actors). Laryngopharyngeal reflux was diagnosed based on the scores of Reflux Symptom Index (RSI) chart [12].

### 2.3. Clinical Types

**Figure 1:**
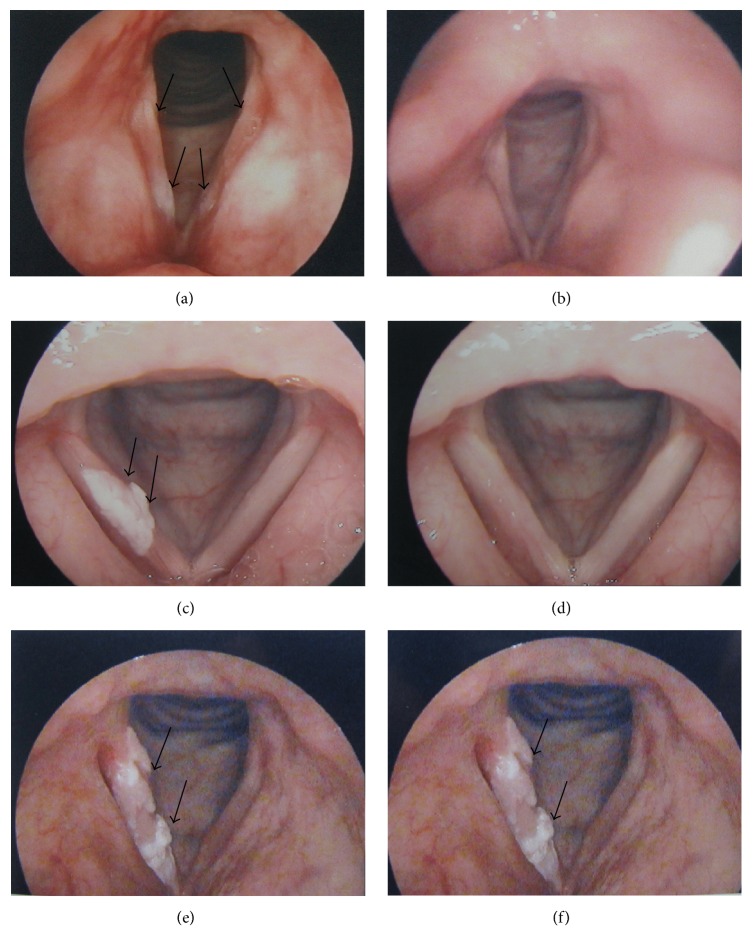
Pretreatment (a) and posttreatment (b) pictures of flat and smooth vocal fold leukoplakia (complete response). Pretreatment (c) and posttreatment (d) pictures of elevated and smooth vocal fold leukoplakia (complete response). Pretreatment (e) and posttreatment (f) pictures of rough vocal fold leukoplakia (no response). Black arrowheads indicate the three types of vocal fold leukoplakia.

Morphological characteristics including surface, margin, and texture were recorded; then vocal fold leukoplakia was subdivided into three categories by three experienced laryngologists independently: flat and smooth, elevated and smooth, and rough (Table 1). Representative photos of each lesion type are shown in Figure 1. When vocal fold leukoplakia lesion had more than one morphological appearance on different locations, the lesion was categorized as elevated and smooth type if flat and smooth leukoplakia and elevated and smooth leukoplakia coexisted on vocal cords; rough leukoplakia was determined once rough lesion appeared on vocal cords.

### 2.4. Treatment

Patients with rough leukoplakia were strongly recommended for vocal fold mucosal stripping by carbon dioxide (CO_2_) laser. Nonsurgical treatment was conducted for patients with smooth vocal fold leukoplakia or patients with rough leukoplakia who had high-risk medical problems in surgery or strongly required to receive conservative treatment.

Nonsurgical treatments included smoking and drinking cessation, strict voice rest, proton pump inhibitor (omeprazole 20 mg twice daily) therapy if accompanied with laryngopharyngeal reflux, and Chinese medication (Xuanboshuangsheng Granules 8 g twice daily). The main ingredients of Xuanboshuangsheng Granules are herbs including Radix Scrophulariae, Cortex Phellodendri, and Radix Glycyrrhizae (Drug Approval Number: Z05170495, Shanghai, China; Medical Institution: Ear, Nose, Throat Hospital of Fudan University, Shanghai, China; Associated Institution: Shanghai Liantang Pharmaceutical Corporation Limited).

Continuous nonsurgical therapy lasted for 6 weeks. Patients were seen at 2- to 4-week intervals in the first 3 months and 4- to 12-week intervals in the following months and evaluated by office-based laryngoscopic examination. Vocal fold mucosal stripping by CO_2_ laser was performed for patients who had no improvements to previous nonsurgical treatment after a follow-up of 6 months.

### 2.5. Clinical Response Evaluation

The laryngoscopic images of each patient before and after treatment were compared by three experienced laryngologists. Complete response (CR) was defined as complete disappearance of the lesion for at least 4 weeks. Partial response (PR) was defined as reduction in lesion size of 50% or more for at least 4 weeks. No response (NR) was defined as no significant change for at least 4 weeks, including stable disease, reduction of less than 50%, and lesions with increase of less than 25%. Progressive disease (PD) was defined as appearance of any new lesions not previously identified or estimated increase of 25% or more in existent lesions or the progression from smooth lesion to rough lesion [13]. The time to complete response of patients was the time from patient's initial visit until complete disappearance of lesion.

### 2.6. Histological Assessment

All the tissues were routinely processed for pathological examination. Formalin-fixed and paraffin-embedded slides were independently viewed and histologically graded by three pathologists in the Department of Pathology at Eye and Ear, Nose, Throat Hospital of Fudan University, Shanghai, China. Epithelial dysplasia was determined according to the World Health Organization 2005 classification in which vocal fold leukoplakia is divided into the following categories: squamous cell hyperplasia with nondysplasia, mild dysplasia, moderate dysplasia, severe dysplasia, and carcinoma [14]. Squamous cell hyperplasia with nondysplasia describes increased cell numbers but the architecture shows regular stratification and there is no cellular atypia. Mild dysplasia describes slight cytological atypia, most marked in the basal one-third of the epithelium. Moderate dysplasia describes more cytological atypia, changes presenting in the lower two-thirds of the epithelium. Severe dysplasia describes cytological atypia involving more than two-thirds of the epithelial thickness. Carcinoma describes full thickness architectural abnormalities in the viable cellular layers accompanied with cytologic atypia.

### 2.7. Statistical Analysis

All statistical analyses were performed using SPSS software version 23.0 (IBM Corporation, 2015, USA). All comparisons of clinical data among three groups were by Fisher's exact test except age (one-way analysis of variance). Fisher's exact test was conducted to evaluate clinical response of three groups. Then pairwise comparisons were conducted among three groups using Bonferroni's test. The complete response of smooth types was also presented as Kaplan-Meier curves with statistical comparison using log-rank test. The ordinal logistic regression model was conducted to determine distinct clinical factors affecting clinical response. Fisher's exact test and Kruskal-Wallis test followed by Nemenyi test were used to evaluate relationship between pathological grades and clinical types. Two-tailed *P* values < 0.05 were statistically significant for analysis except Bonferroni's test (*P* values < 0.017).

## 3. Results

### 3.1. Characteristics of Patients

A total of 178 patients with vocal fold leukoplakia treated with nonsurgical therapy were included in this study. The characteristics of baseline patient information are showed in Table 2. Of these case, 66 (37.1%) showed flat and smooth leukoplakia, 103 (57.9%) showed elevated and smooth leukoplakia, and 9 (5%) showed rough leukoplakia. 171 (96.1%) patients were male and only 7 (3.9%) patients were female, whose average age was 49.8 ± 8.9 years (ranging from 28 to 73 years). The mean age of the patients with rough leukoplakia was significantly older than the patients with smooth leukoplakia (rough versus flat and smooth, *P* < 0.001; rough versus elevated and smooth group, *P* < 0.001). Otherwise, the groups were well balanced.

### 3.2. Clinical Response after Nonsurgical Treatment

Clinical response of patients with vocal fold leukoplakia is showed in Table 3. The complete response rate among three groups was significantly different (*P* < 0.001, Fisher's exact test). Following pairwise comparisons, patients with smooth leukoplakia had a significantly higher complete response rate than those with rough leukoplakia (flat and smooth versus rough, OR, 0.20, 95% CI, 0.12 to 0.32, *P* < 0.001, Fisher's exact test; elevated and smooth versus rough, OR, 0.34, 95% CI, 0.26 to 0.45, *P* < 0.001, Fisher's exact test). Complete response rate of flat and smooth leukoplakia and elevated and smooth leukoplakia had no significant difference (OR, 0.48, 95% CI, 0.23 to 0.99, *P* = 0.055, Fisher's exact test). Appearance of clinical response for vocal fold leukoplakia is showed in Figure 1.

**Figure 2:**
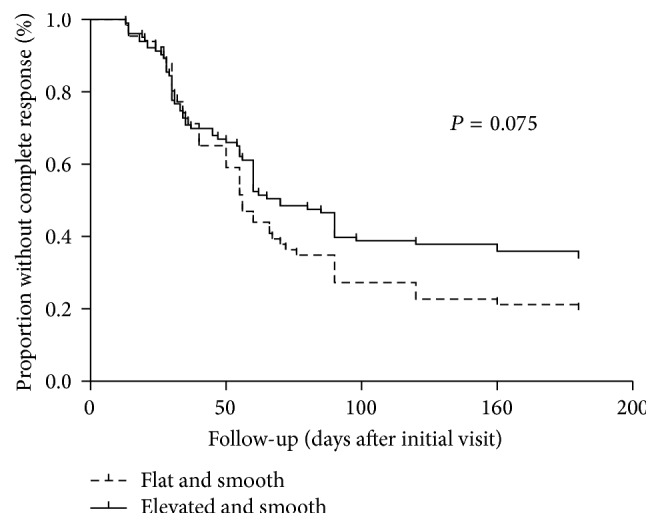
Kaplan-Meier survival curves for complete response of patients with smooth vocal fold leukoplakia.

The mean time to complete response was 55.3 ± 38.3 days of two smooth types. Complete response rate of smooth types was subsequently estimated by Kaplan-Meier survival curves and compared using log-rank test. Kaplan-Meier analysis showed that the 3-month complete response rate was 72.7% for flat and smooth type compared with 60.2% for elevated and smooth type. After 6 months, complete response rates were 80.3% and 66.0%, respectively (*P* = 0.075, log-rank test; Figure 2).

The ordinal logistic regression analysis showed that clinical type (*P* = 0.005) was the only significant variable that influenced the clinical response. Other factors, including gender, age, smoking, alcohol consumption, laryngopharyngeal reflux, voice abuse, and site of lesions, appeared to be insignificant with clinical response (Table 4).

### 3.3. Postoperative Pathological Results

A total of 51 patients who had no improvements to nonsurgical therapy after a follow-up of 6 months received surgery. Of these cases, 11 (21.6%) showed flat and smooth leukoplakia, 31 (60.8%) showed elevated and smooth leukoplakia, and 9 (17.6%) showed rough leukoplakia. The pathological results of vocal fold leukoplakia are listed in Table 5. The incidence of carcinoma in rough leukoplakia was significantly higher than that in smooth leukoplakia (44.4% versus 2.4%, OR, 32.8, 95% CI, 3.04 to 354.4, *P* = 0.002, Fisher's exact test). The correlation coefficient of three morphological groups and pathological grades was *P* < 0.001. The result of Kruskal-Wallis test followed by Nemenyi test noted that statistical significant differences between rough leukoplakia and smooth leukoplakia were observed, respectively (rough versus flat and smooth, *P* < 0.001; rough versus elevated and smooth, *P* = 0.008).

## 4. Discussion

Vocal fold leukoplakia can be histologically diagnosed as squamous cell hyperplasia, mild dysplasia, moderate dysplasia, severe dysplasia, and carcinoma according to pathological classification systems [14]. However, there have been few reports about clinical classification of vocal fold leukoplakia. Oral leukoplakia was divided into two subtypes as nonhomogenous and homogenous [8]. Lee et al. divided vocal fold leukoplakia into three morphological groups: superficial type, exophytic type, and ulcerative type [15]. Fang et al. proposed a method to categorize the vocal fold leukoplakia based on morphologic characteristics scoring, which included thickness, texture, color, hyperemia, size, and symmetry [9]. Similarly, this study proposed a new morphological classification of vocal fold leukoplakia. In the last decades, new endoscopic tools, especially narrow band imaging, have been used for clinical classification of vocal leukoplakia based on microvascular changes [16], whereas the present classification according to macroscopic appearance provides a valuable source of laryngoscopic examination, which is more commonly applied in clinical practice.

There is still no agreement on the management of vocal fold leukoplakia. To data, surgical treatment has been suggested as an option [1, 4]. Although the disappearance and reduction of oral leukoplakia with nonsurgical therapy have been documented in the past [17, 18]. To our knowledge, there have been few records about the effectiveness of nonsurgical therapy for vocal fold leukoplakia. Xu et al. found that a complete response up to 85% was observed in vocal leukoplakia with andrographolide therapy with a follow-up of 12 months [19]. In our study, 127 of 178 patients (71.3%) with nonsurgical treatment had complete or partial response. Additionally, we analyzed the time to complete response (mean ± SD, 55.3 ± 38.3 days) of nonsurgical treatment in vocal fold leukoplakia for the first time. These findings demonstrated that some lesions of vocal fold leukoplakia might disappear or decrease in size without surgical therapy and these lesions might benefit from nonsurgical intervention.

A study of oral leukoplakia without surgical treatment demonstrated 32.5% of homogenous lesions and 24.3% of nonhomogenous lesions, respectively, disappeared or reduced [20]. Likewise, the analyses of data (Table 3) noted that the effectiveness of smooth leukoplakia was better in comparison to rough leukoplakia. Nonsurgical treatment exhibited significant curative effects to smooth leukoplakia. Result of Kaplan-Meier analysis noted that there was no significant difference of complete response rate for two smooth types. Elevated and smooth leukoplakia behaves similar to flat and smooth leukoplakia and therefore should be managed similarly.

The risk factors including tobacco smoking, alcohol intake, voice abuse, and laryngopharyngeal reflux might be related to vocal fold leukoplakia [14]. It was reported that tobacco smoking is the most important factor that could increase the disappearance of oral leukoplakia [17]. However, we made comprehensive analysis based on various clinical factors including clinical type, gender, age, smoking, alcohol use, voice abuse, laryngopharyngeal reflux, and site of lesions. The only significant factor associated with clinical response was clinical type of vocal fold leukoplakia. Based on our multivariate analysis regression models, patients who present with a smooth vocal fold leukoplakia would best be served by nonsurgical treatment and patients who present with a rough vocal fold leukoplakia would need aggressive therapy. Additionally, it remains unknown whether pathological grade of vocal fold leukoplakia would affect the clinical response of nonsurgical treatment. In the present study, this issue on vocal fold leukoplakia is unable to be investigated since pathological results cannot be determined without a biopsy which might deteriorate the quality of voice.

Vocal fold leukoplakia should be managed on its benign and malignant possibilities. Hyperplasia with nondysplasia or mild dysplasia could not be regarded as a precancerous lesion of larynx and should be managed with no surgical intervention; the lesion with more than moderate dysplasia should be managed more aggressively [5, 21, 22]. In the present study, vocal fold mucosal stripping by CO_2_ laser was performed in 51 patients who showed no improvements upon previous nonsurgical treatment. The results of the pathological diagnosis showed that smooth lesions mainly presented with nondysplasia and mild dysplasia in pathology, whereas rough lesions mainly presented with severe dysplasia and carcinoma. We believed that this classification method was useful for differentiating between benign and malignant lesions. Additionally, following clinical data of three groups compared (Table 2), the mean age of the patients with rough leukoplakia was significantly older than those with smooth leukoplakia; therefore, age was an important factor to consider when we identify vocal fold leukoplakia types appropriate for nonsurgical treatment based on this classification method.

The first limitation of this study is lack of a control group of patients receiving surgery. Secondly, the number of patients with rough leukoplakia was only 9 due to our recommendation that patients with rough leukoplakia should be treated with surgery in consideration of malignant risk. Thirdly, the relationship between clinical type and pathological grades needs to be studied with a larger sample size. Lastly, a prospective cohort study is required to validate the usage of the classification method and recognize the effect of nonsurgical treatment.

## 5. Conclusion

The effectiveness of nonsurgical treatment for smooth vocal fold leukoplakia is significantly better in comparison to rough vocal fold leukoplakia. The classification method is recommended to guide the decision-making about indications for management. In general, smooth leukoplakia could be managed with nonsurgical treatment; more aggressive treatments should be considered for rough leukoplakia.

## Figures and Tables

**Table 1:** Morphological classification of vocal fold leukoplakia.

Clinical type	Definition
Flat and smooth type	Surface: smooth Margin: lesion without raised margins, being continuous with the surrounding mucosa Texture: homogenous and regular, the lesion having even coloration

Elevated and smooth type	Surface: smooth Margin: lesion with raised margins, sharply demarcated from the surrounding mucosa Texture: homogenous and regular, the lesion having even coloration

Rough type	Surface: wrinkled, corrugated Margin: lesion with raised margins, sharply demarcated from the surrounding mucosa Texture: nonhomogenous and irregular, the lesion having uneven coloration, usually accompanied with erosion or ulceration

**Table 2:** Characteristics of baseline patient information.

	Flat and smooth	Elevated and smooth	Rough	*P* ^*∗*^
Gender				
Male	63	99	9	0.675
Female	3	4	0	
Age	48.7 ± 8.5	49.3 ± 8.4	63.3 ± 7.9	<0.001
Smoking				
Yes	48	87	8	0.145
No	18	16	1	
Alcohol consumption				
Yes	27	46	1	0.132
No	39	57	8	
Laryngopharyngeal reflux				
Yes	13	20	1	0.503
No	53	83	8	
Voice abuse				
Yes	58	90	7	1.000
No	8	13	2	
Site of lesion				
Unilateral vocal cord	21	44	6	0.089
Bilateral vocal cords	45	59	3	

^*∗*^All comparisons were by Fisher's exact test except age (one-way analysis of variance).

**Table 3:** Clinical response of three types for vocal fold leukoplakia.

	Complete response		Total
	Yes	No	
Flat and smooth	53 (80.3%)	13 (19.7%)	66 (37.1%)
Elevated and smooth	68 (66.0%)	35 (34.0%)	103 (57.9%)
Rough	0 (0.0%)	9 (100.0%)	9 (5%)
Total	121 (68.0%)	57 (32.0%)	178 (100%)

**Table 4:** Relationship between clinical response and clinical characteristics.

	CR	PR	NR	PD	Total	*P*
Clinical type						
Smooth	121	6	34	8	169	0.005
Rough	0	0	8	1	9	
Gender						
Male	115	6	41	9	171	0.581
Female	6	0	1	0	7	
Age						
<60	111	5	33	7	156	0.347
≥60	10	1	9	2	22	
Smoking						
Yes	94	6	35	8	143	0.396
No	27	0	7	1	35	
Alcohol consumption						
Yes	56	3	12	4	74	0.447
No	65	3	30	5	104	
Laryngopharyngeal reflux						
Yes	27	1	5	1	33	0.147
No	94	5	37	8	145	
Voice abuse						
Yes	105	6	37	8	155	0.535
No	16	0	5	1	23	
Site of lesions						
Unilateral vocal cord	43	3	19	6	71	0.187
Bilateral vocal cords	78	3	23	3	107	

CR, complete response; PR, partial response; NR, no response; PD, progressive disease.

**Table 5:** Pathological diagnosis of vocal fold leukoplakia.

	Nondysplasia	Mild dysplasia	Moderate dysplasia	Severe dysplasia	Carcinoma
Flat and smooth	8	2	1	0	0
Elevated and smooth	8	11	8	3	1
Rough	0	1	1	3	4
Total	16	14	10	6	5
